# Monitoring and analysis of ground subsidence in Shanghai based on PS-InSAR and SBAS-InSAR technologies

**DOI:** 10.1038/s41598-023-35152-1

**Published:** 2023-05-17

**Authors:** Zhihua Zhang, Changtao Hu, Zhihui Wu, Zhen Zhang, Shuwen Yang, Wang Yang

**Affiliations:** 1 Faculty of Geomatics, Lanzhou Jiaotong University, Lanzhou, 730070 China; 2 National-Local Joint Engineering Research Center of Technologies and Applications for National Geographic State Monitoring, Lanzhou, 730070 China; 3 Gansu Province Engineering Laboratory for National Geographical State Monitoring, Lanzhou, 730070 China

**Keywords:** Geology, Environmental sciences

## Abstract

Shanghai is susceptible to land subsidence due to its unique geological environment and frequent human activities. Traditional leveling techniques are not sufficient for monitoring large areas of land subsidence due to the time-consuming, labor-intensive, and expensive nature of the process. Furthermore, the results of conventional methods may not be timely, rendering them ineffective for monitoring purposes. Interferometric Synthetic Aperture Radar (InSAR) technology is a widely used method for monitoring ground subsidence due to its low cost, high efficiency, and ability to cover large areas. To monitor the surface sink condition of Shanghai over the past 2 years, monitoring data were obtained through the technical processing of 24 images from Sentinel-1A data covering Shanghai from 2019 to 2020 using the Persistent Scatterer (PS-InSAR) and Small Baseline Subset (SBAS-InSAR) technique. The ground subsidence (GS) results were extracted via PS and SBAS interferometry processing, while Shuttle Radar Topography Mission data were used to correct the residual phase. According to PS and SBAS methods, the maximum ground subsidence in the study area reached 99.8 mm and 47.2 mm, respectively. The subsidence rate and the accumulated amount of subsidence derived from the monitoring results revealed the urban area in Shanghai to be principally characterized by uneven GS, with multiple settlement funnels being found to be distributed across the main urban area. Moreover, when compared with the historical subsidence data, geological data, and urban construction distribution data, the individual settlement funnels were observed to correspond to those data concerning the historical surface settlement funnel in Shanghai. By randomly selecting GS time-series data regarding three feature points, it was determined that the morphological variables of the GS remained largely consistent at all time points and that their change trends exhibited a high degree of consistency, which verified the reliability of the PS-InSAR and SBAS-InSAR monitoring method. The results can provide data support for decision making in terms of geological disaster prevention and control in Shanghai.

## Introduction

Ground subsidence is a geological phenomenon associated with the slow descent of the earth due to a variety of factors (e.g., geological environment, surface load, influence of human elements, etc.)^1–3^. It is recognized as a slowly changing geohazard with irreversible characteristics and long-term impacts^4–7^. Ground subsidence can directly cause serious damages to housing areas, transportation networks, industrial areas, agriculture lands, and other infrastructures and indirectly result in consequences including increase of flooding extent, variations in soil–vegetation properties, and so on^8–13^. It not only seriously affects the urban planning and construction of Shanghai, but also increases the risk of land salinization, seawater intrusion, and backward irrigation. On July 5, 2017, the parking lot of a hotel in Pudong collapsed, with the ground splitting more than 20 m; on September 21, 2018, a severe ground collapse occurred on Yunling West Road in Putuo, Shanghai, caused a passing taxi to sink into it^6,7^. Recently, such accidents have frequently occurred, damaging the safety of people’s lives and property. It is further estimated that the economic loss caused by ground subsidence in Shanghai from 2001 to 2020 reached 24.57 billion yuan higher. Therefore, it is imperative to monitor the surface subsidence in the urban areas of Shanghai.

According to historical data^6,14^, the maximum accumulated amount of surface settlement in some areas of Shanghai has now reached 1.69 m. A high surface settlement rate can result in significant harm to buildings and people’s living environments. Over the course of the twenty-first century to date, the average annual surface settlement rate in Shanghai has accelerated, while uneven surface settlement has occurred in many areas^15,16^. Traditional ground subsidence measurement methods include the level survey, bedrock marker survey, and stratified marker survey methods^7,17^. However, such methods can only obtain subsidence information at discrete points on the ground, which means that they cannot be used to perform dynamic monitoring over a large area^18–21^.

Traditional land subsidence monitoring methods are costly, time consuming, and limited in range^22–25^. Interferometric synthetic aperture radar (InSAR) has the advantages of high spatial and temporal resolution, high monitoring accuracy, and continuous observation^26–28^. By mining time series SAR images using PS-InSAR^29–31^, and SBAS-InSAR (i.e., a small baseline subset of InSAR)^32,33^, accuracy can reach the submillimeter level^34,35^. With the increasing enrichment of space-borne SAR data, InSAR has become an important technical mean of monitoring land subsidence^36^. The InSAR technique, independent of time and weather, can monitor small deformations on the ground in near-real-time over a large area. The permanent scatterer interferometry technique proposed by Ferretti et al.^30^ and Zhou et al.^37^, is one of the Time-series InSAR (TS-InSAR), which is more effective for monitoring subsidence in urban areas and can reach millimeter-level accuracy.

TS-InSAR technology is increasingly used in urban surface settlement monitoring. Gao et al.^20^ processed 23 scenes of Sentinel-1A images from 2015 to 2017 by SBAS-InSAR and PS-InSAR to obtain surface subsidence data based on the two methods in the urban area of Nanjing. Finally, the two sets of data are used as a check and corroboration for each other to analyze the results. It showed that the results obtained by the two methods were in high agreement. Ma et al.^38^ used PS-InSAR to monitor the settlement in Tianjin. They compared it with the level measurement results to prove the reliability of PS-InSAR in urban surface settlement monitoring results, which can reach millimeter-level accuracy. Liu et al.^39^ used PS-InSAR and SBAS-InSAR techniques to monitor surface subsidence in Jining city, respectively. They then analyzed and compared the subsidence results obtained from the two technical methods to verify their applicability in urban surface monitoring based on accuracy and precision^40,41^. In many large cities, over-exploitation of groundwater is also the main cause of land subsidence, Khorrami et al.^42^ and Mahmoodinasab et al.^43^ used PS-InSAR technology to effectively monitor the land subsidence, and expounded the direct relationship between groundwater exploitation and ground subsidence. In addition, the construction of underground facilities such as subways is another major factor leading to urban land subsidence, InSAR technology plays an important role in obtaining reliable ground subsidence monitoring results^44,45^. The PS-InSAR and SBAS-InSAR method can be applied to urban surface settlement monitoring with high accuracy.

PS-InSAR is a method that analyzes point targets, primarily used for analyzing scattering objects that exhibit high coherence. This method derives accurate height measurements and linear displacements related to local scattering objects, typically characterized by high coherence. The quantity of SAR data is crucial for estimating coherence and identifying stable PS points. Insufficient data continuity can result in overestimating coherence, leading to numerous false PS points, and incorrect deformation results^46^. To obtain reliable PS points and results, at least 20 temporal datasets are required, with regular, continuous, and available acquisition times, ideally once a month. PS technology is suitable for urban areas or areas with relatively stable interference and radiation ratios.

In addition, The SBAS-InSAR method^47,48^ is suitable for analyzing distributed targets. The results of SBAS are similar to those of traditional D-InSAR processing results, derived by using more SAR data in time series. Compared to PS, SBAS requires fewer data in the temporal domain because it calculates based on the spatial distribution of coherence, rather than considering only individual pixels. In general, the more temporal datasets, the better the results, as more data aids in estimating and removing atmospheric phases. SBAS has three deformation models available: linear, quadratic, and cubic models, in addition to a no-deformation model, which can be used to extract DEM. In the absence of temporal constraints, due to phase unwrapping, the maximum displacement relative to spatial variations is limited. Additionally, SBAS is more versatile than PS, as it can utilize all available interferometric pairs. For measuring deformation types, PS is only applicable for linear deformation, while SBAS is applicable for both linear and non-linear deformation.

Inspired by the above analysis, we adopt PS-InSAR and SBAS-InSAR for settlement monitoring in Shanghai, obtaining data for a subsidence rate map and DEM, which will provide a basis for the future study’s assessment of subsidence disaster risk^49^.

## The study area and data source

Shanghai is located in the southeast region of the Yangtze River Delta, with a large area of quaternary loose sediments. Only a tiny amount of bedrock is exposed in the southwest area. Most regions of Shanghai have flat terrain and low altitude, generally at 2–6 m. Due to the riverside and seaside, Shanghai has low and flat terrain, a relatively fragile geological environment, soft soil is widely distributed, high groundwater level and the distribution of the aquifer system is relatively complex. The excessive exploitation of groundwater is a major factor leading to ground subsidence. In recent years, the underground space development activities represented by the deep foundation pit project have significantly disturbed the shallow soft soil in Shanghai, which has become another major contributing factor to ground subsidence^6^.

After consulting relevant literature^6^, it was discovered that Shanghai's maximum groundwater extraction occurred in 1993, with a total of 153 × 10^6^ m^3^ extracted, this caused severe ground subsidence. Measures have been implemented to manage and control the extraction, resulting in a decrease to less than 5 × 10^6^ m^3^ by 2014. In contrast, the amount of water recharged has increased to 25 × 10^6^ m^3^. Since 2010, the amount of water recharged has exceeded the amount of water extracted annually. Therefore, it can be concluded that ground subsidence after 2014 is primarily caused by human activities rather than groundwater extraction. Consequently, the effects of groundwater extraction on ground subsidence would no longer be analyzed.

Shanghai Metro is the longest urban rail transit system in the world, serving Shanghai and the surrounding metropolitan area in China. As of December 2021, it consists of 19 lines (excluding maglev lines) and 508 stations, with a total length of 831 km. Given the substantial construction involved in the development of the metro system, it is important to consider its potential impact on ground subsidence. Therefore, we will focus on analyzing the impact of Shanghai Metro on ground subsidence.

**Figure 1:**
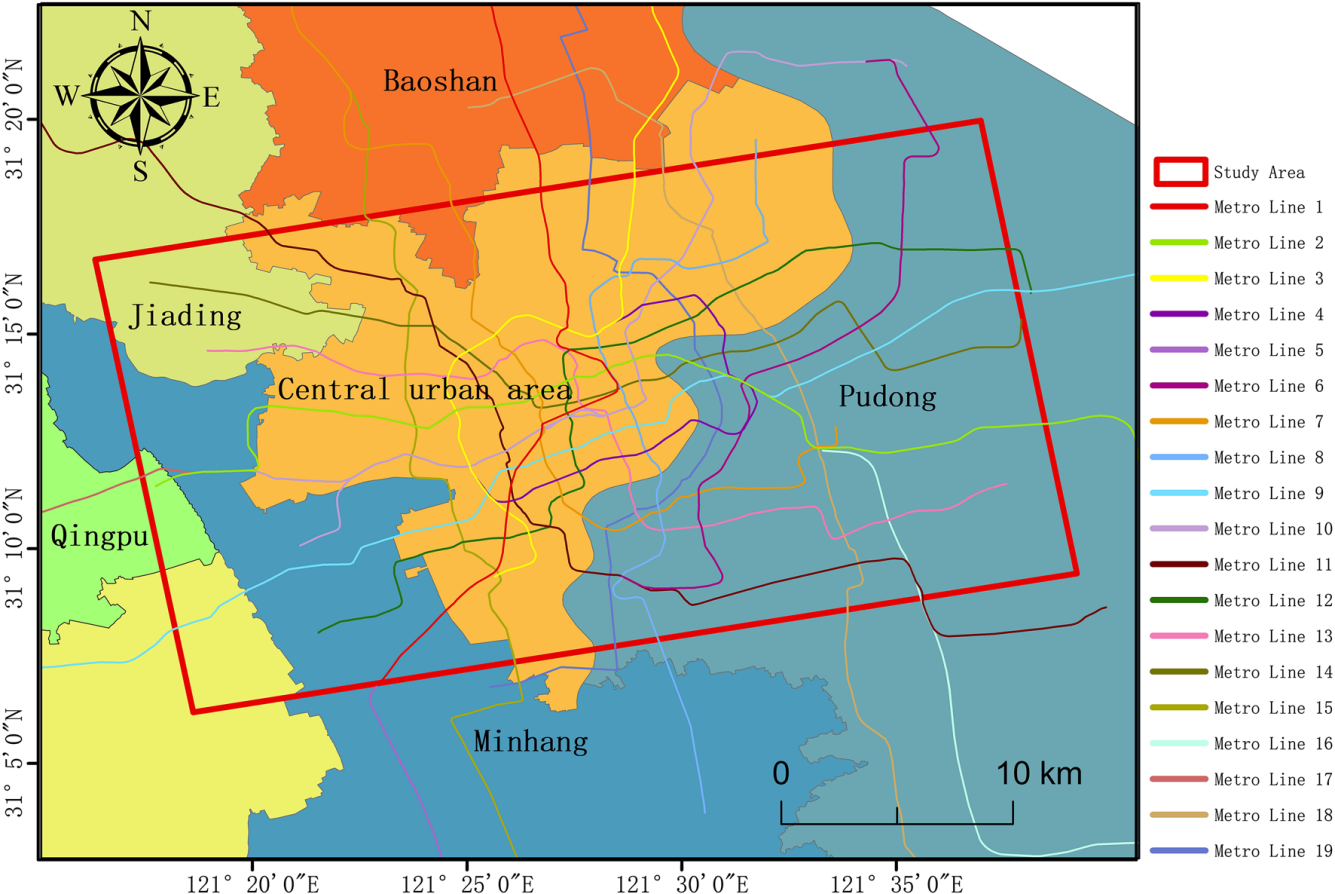
Geographical location of the study area. The data has been acquired from the Baidu Atlas. The map and the metro data are visualized and integrated with ArcMap 10.5.0, http://www.esri.com.

In Shanghai, from January 2019 to December 2020, we used 24-images Sentinel-1 A data for urban GS monitoring, which we downloaded from the Alaska Satellite Center (https://vertex.daac.asf.alaska.edu/#). The Sentinel-1A data is different from ordinary remote sensing data in that its SAR coordinates and geographical coordinates are reversed. For the elevation data, we used SRTM1 data with a resolution of 30 m × 30 m. The research area included the center of Shanghai and parts of the Pudong district, Minhang district, Jiading district, Baoshan district, Qingpu and Songjiang districts, Yangpu district, Hongkou district, Changning district, Xuhui district, Jing’an district, and Putuo district. The specific geographical location is shown in Fig. 1.

## PS-InSAR processing

PS-InSAR^50–52^ mainly analyzes the change state for surface point targets, and the result is a large number of vector points, each of which contains information about the surface deformation. According to its technical process specification, in order to ensure the high precision and accuracy of its technical processing results, it is recommended to use at least 20 scenes of multi-temporal data, and the time between two scenes of data before and after 20 scenes needs to have continuity, and the time interval should not be too large (the best one scene of data around (within) 1 month). The PS-InSAR technique is suitable for urban surface monitoring because there are a large number of buildings in the city, most PS points are chosen in the fixed building corners (permanent scatterers) of the city during the technical processing. The PS-InSAR technique can monitor the displacement at the millimeter level and extrapolate the surface deformation rate with the help of time series from multi-temporal data.

### Generating connection diagrams

**Figure 2:**
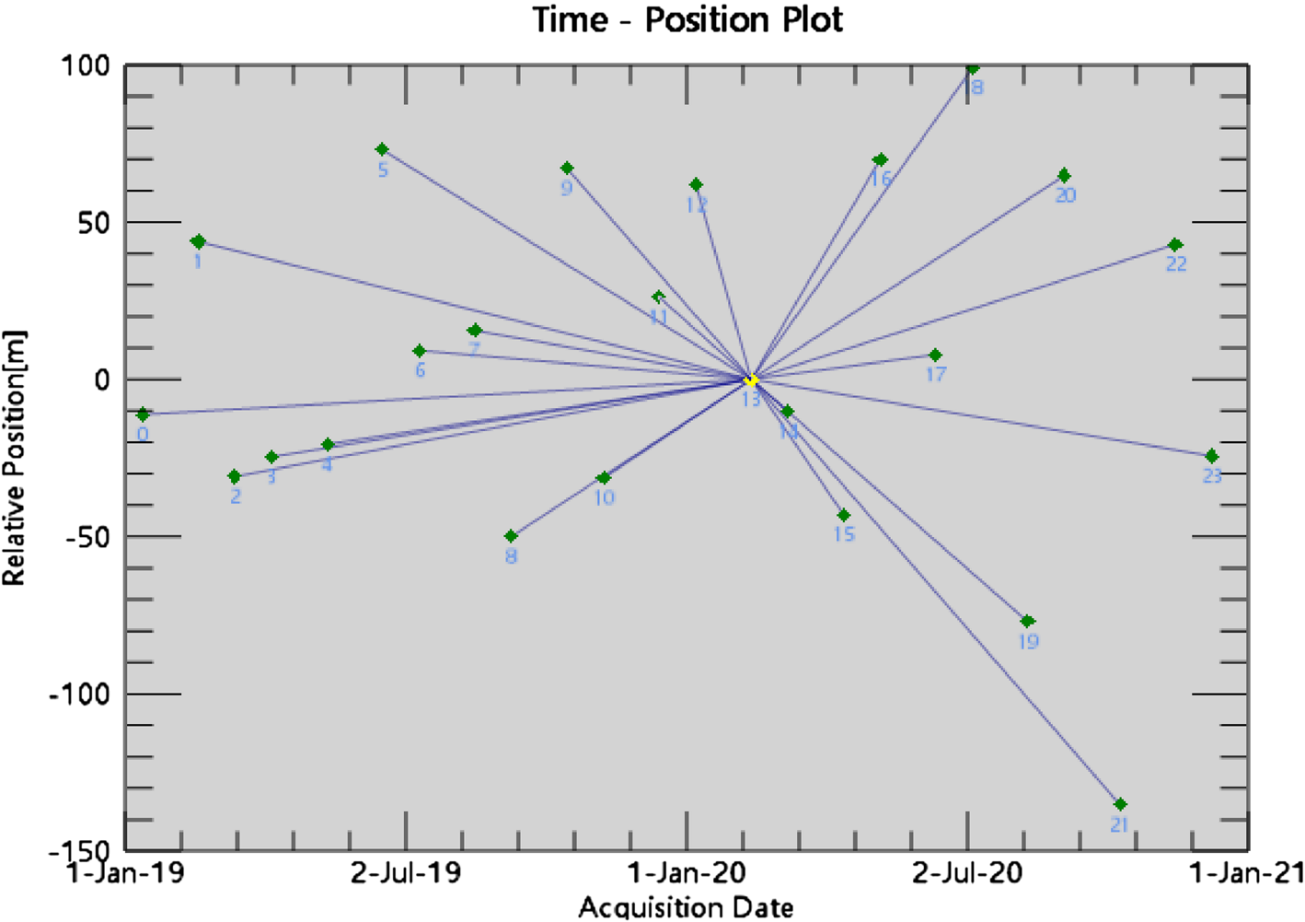
Connection diagram.

The data pairs and linkage maps are generated by linking the multi-temporal Sentinel-1A dataset. This experiment uses 24 images from the period 2019–2020 obtained from the Sentinel-1A data covering the Shanghai area. The correlation of InSAR interferograms is mainly influenced by the temporal baseline, spatial baseline, and Doppler centroid frequency baseline. Therefore, the selected image should achieve the optimal combination of these three baselines, that is, to maximize the sum of coherence coefficients in the time series interferograms. Among the 24 images considered, we choose the image with the highest correlation with the other images as the master image. A total of 23 interferometric pairs are generated, with the image from February 12, 2020 serving as the master image. As shown in Fig. 2, the spatial and temporal baseline connection diagrams in which the master image represents the data corresponding to the yellow dot and the qualified image represents the data corresponding to the green dot are successfully paired. Figure 2 indicates that the image data selected for this experiment are all qualified image data.

### Interferometric workflow

The interferometric workflow mainly includes the georeferencing, interferogram generation, deleveling, and amplitude departure index calculation. When georeferencing, the program automatically matches the data (other than the master image) with the master image. The ratio of the distance to the azimuth is set to 6:1 in the parameter settings, which should avoid the issue of rapidly changing interference fringes due to the long baseline. The quality of the deleveling effect is determined based on the accuracy level of the digital elevation model (DEM), which uses processed DEM data from the Shanghai area to perform the interferometric phase deleveling operation.

### First model inversion

The first model inversion is performed to obtain the displacement rate and residual topography, which are then used to de-level the synthetic interferogram. Three types of data are obtained, namely average surface deformation rate data, elevation data (the values are adjusted from the DEM data), and multi-time coherence coefficients.

### Second model inversion

The second model inversion is performed to estimate the atmospheric phase component using the product of the first linear model inversion. The atmospheric phase component is then removed and the final deformation rate is obtained from the second inversion. During the atmospheric estimation process, the dense distribution of the scatterers is used to remove the majority of the propagation delay fluctuations of the signal, while atmospheric filtering is achieved using a combination of high-pass filtering in time and low-pass filtering in space.

### Geocoding

**Figure 3:**
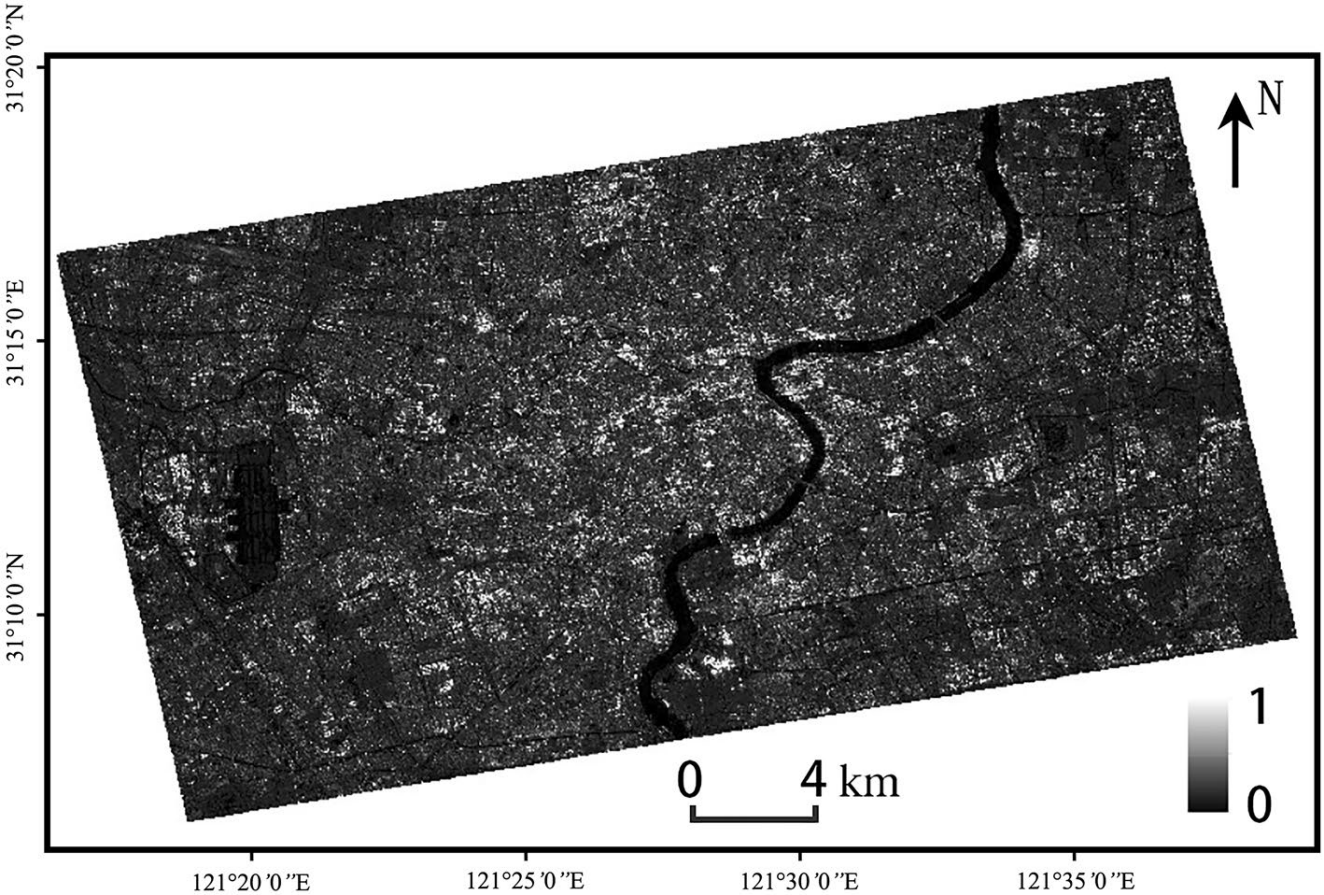
Intensity data. The Sentinel-1A has been acquired from the Alaska Satellite Center, https://vertex.daac.asf.alaska.edu/#. The data are visualized and integrated with SARScape 5.2.1 and ArcMap 10.5.0, http://www.esri.com.

The results of the PS-InSAR processing are geocoded to obtain the intensity data, surface deformation data for the 24 months from January 2019 to December 2020, surface deformation rate data, deformation accuracy data, and elevation accuracy data for 2019 to 2020, as shown in Fig. 3.

**Figure 4:**
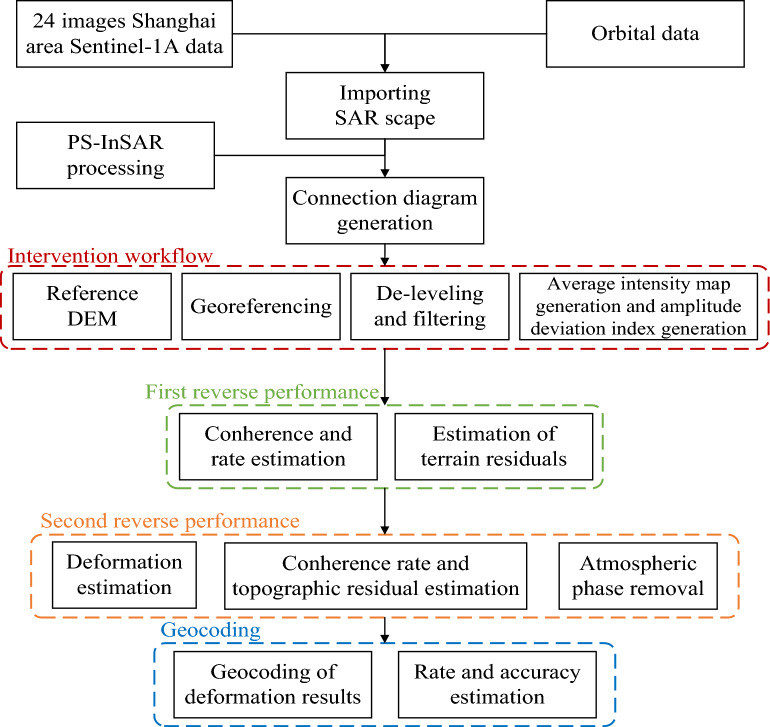
PS-InSAR workflow.

The specific technical flow of the above-described PS data processing method is detailed in Fig. 4.

## SBAS-InSAR data processing

**Figure 5:**
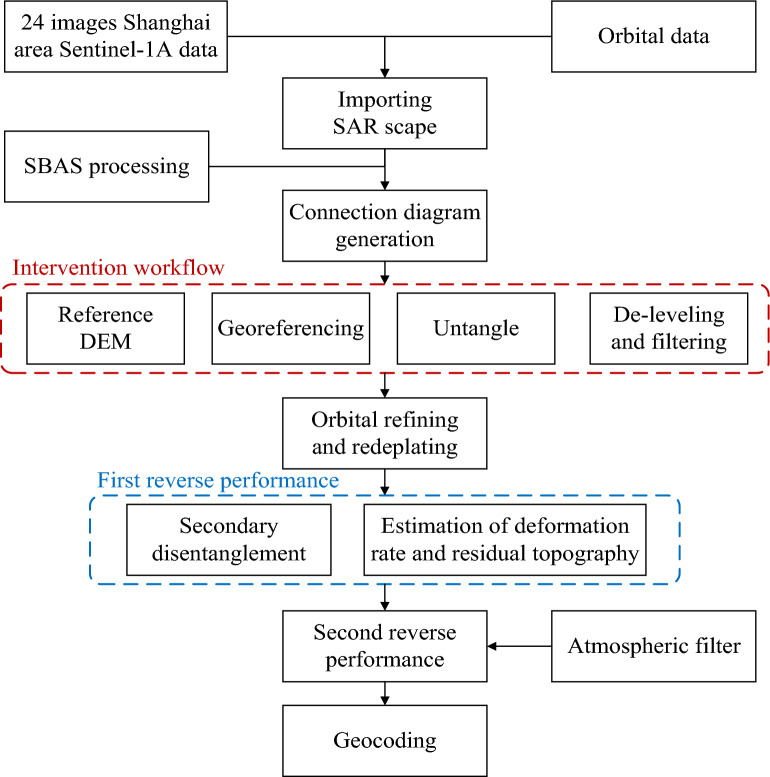
SBAS-InSAR workflow.

There are no level measurement data covering Shanghai during the period of interest with which to verify the results obtained using the PS-InSAR technique on the surface settlement monitoring data covering in the study area. Thus, after processing the SAR data by means of the PS-InSAR technique, the results of same dataset processed by both techniques were compared with each other to check the monitoring results of PS-InSAR technique. The specific technical flow of the SBAS-InSAR technique is detailed in Fig. 5.

### Connection diagrams

**Figure 6:**
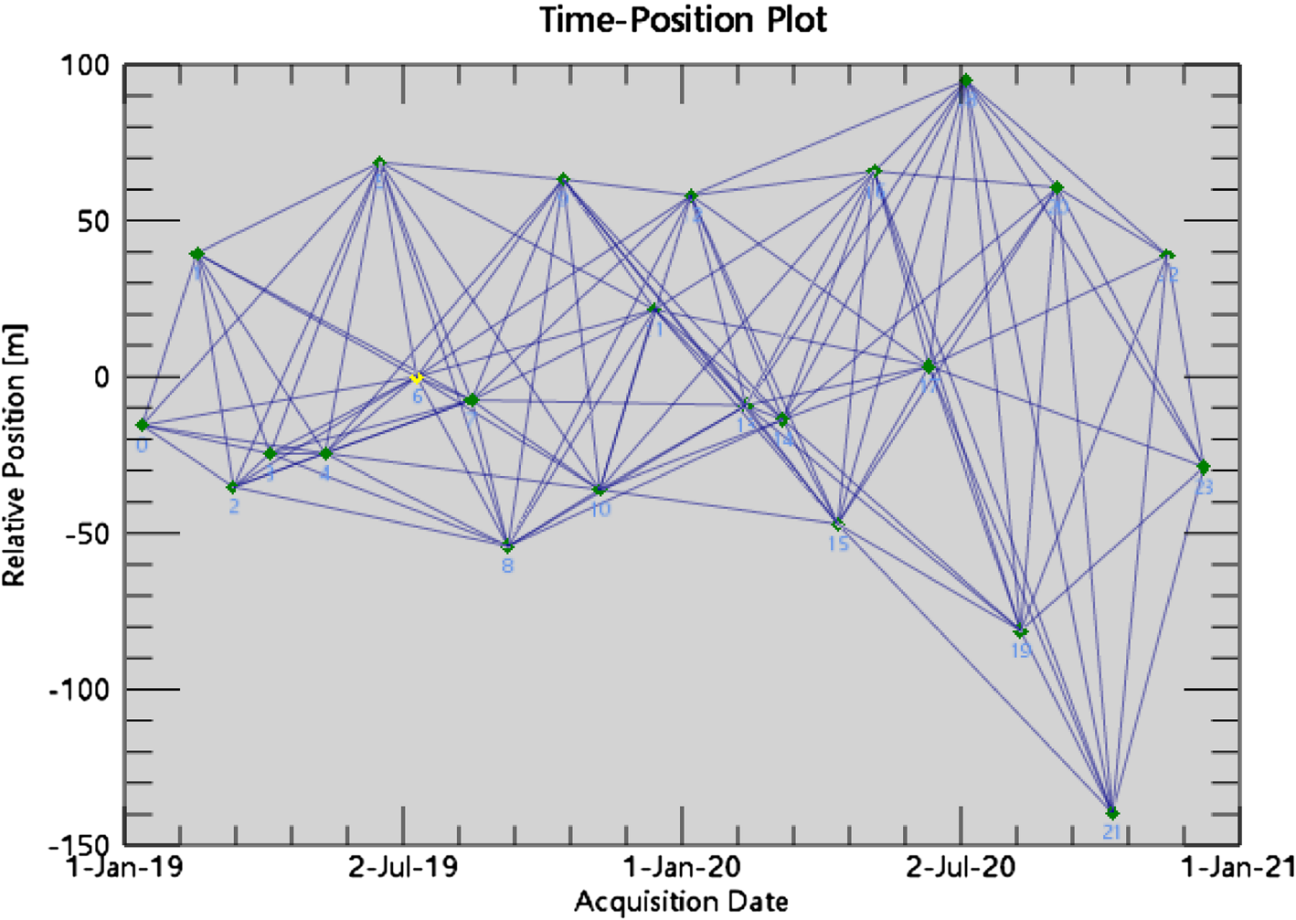
SBAS-InSAR connection diagram generation.

The connection map associated with the SBAS-InSAR technique differs from that associated with the PS-InSAR technique in that data concerning different time phases are freely combined to form the interferometric image pairs. Due to choosing the optimal means of performing the matching and setting the parameter values, 242 pairs of interference image pairs are ultimately generated, as shown in Fig. 6.

### Interference workflow

**Figure 7:**
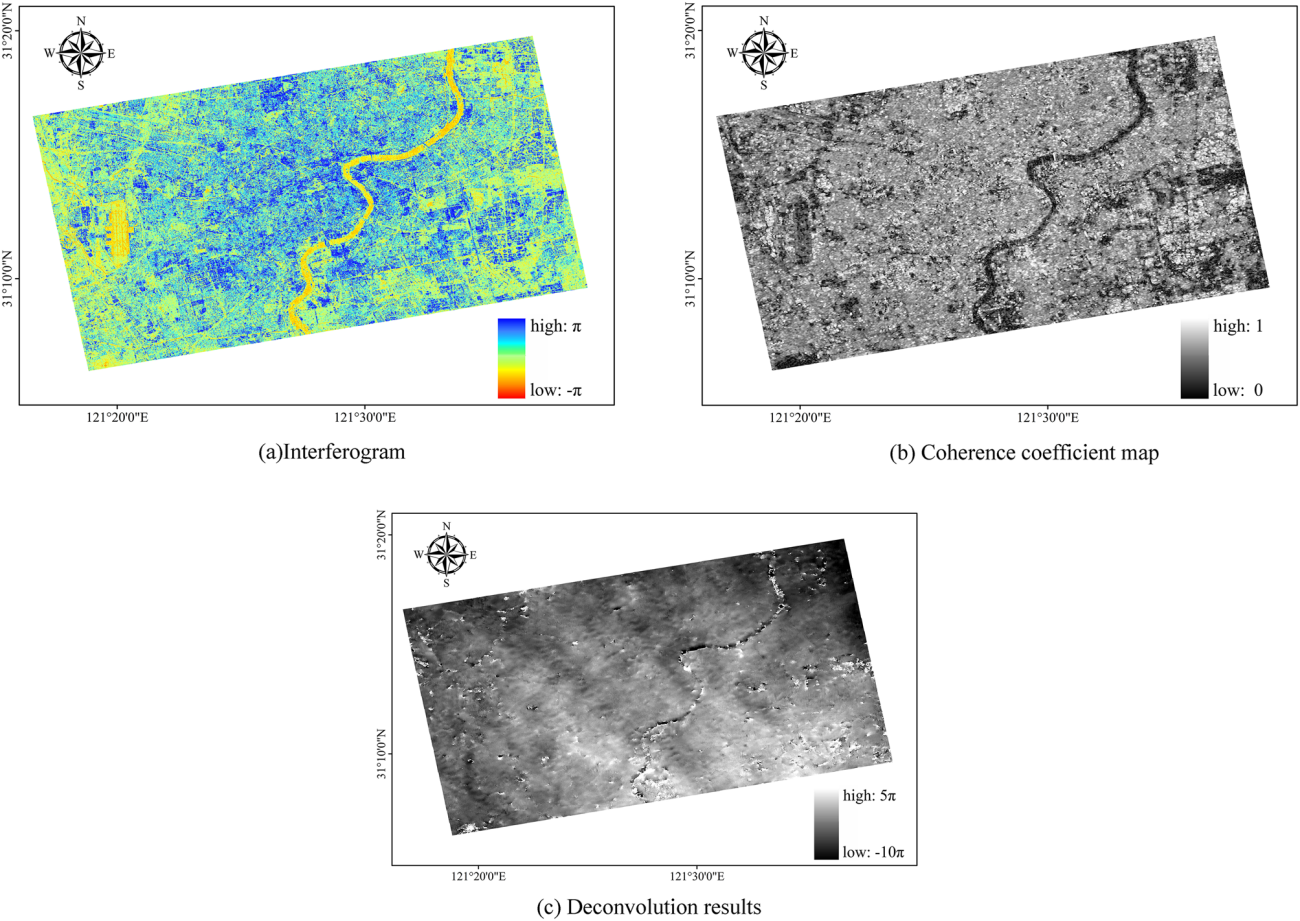
Interference processing results.

The interference workflow includes interferogram generation, interferogram depleting, adaptive filtering and coherence coefficient generation, and phase unwinding, as illustrated in Fig. 7 below. The processing results are filtered and then the multiple interferograms and unwrapping maps are analyzed. If there are any data results with low coherence or poor unwrapping effects, the unqualified image pairs are deleted, as shown in Fig. 7.

### Orbital refining and re-deplating

**Figure 8:**
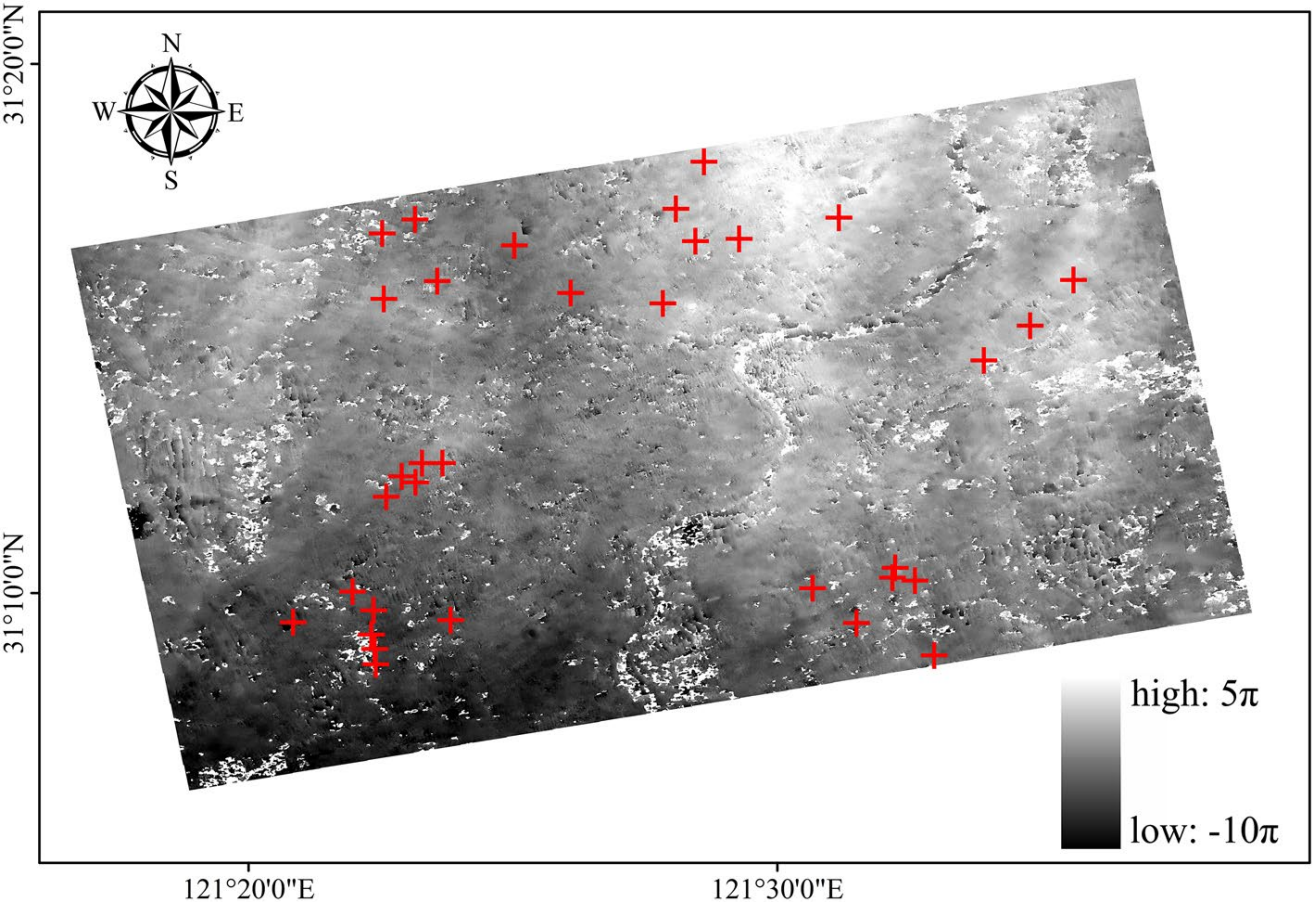
Selecting GCP points.

The orbital refining and re-deplating processes involve the estimation and removal, the residual constant phase and the phase ramp that remains after the unwrapping^26^, which serves to optimize the input data for the inversion process. The re-deplating of all the data is performed using the ground control points (GCPs). When selecting the GCPs, it is important to pay attention to the following. First, there should be no residual topographic stripes or deformation stripes in the selected area, and GCPs should not be selected in an area with deformation. Second, there will be no phase jump, as there will be few suitable areas in the processed results concerning the small baseline subsets, which makes it difficult to identify perfect GCPs. Third, to facilitate the subsequent processing and obtain good data results, more GCPs need to be selected, with at least 30 GCPs being typically required. In this case, according to the demand, 34 GCPs are uniformly selected in the untangled graph, and those points are located in smoother areas to ensure the quality of the GCPs, as shown in Fig. 8.

### First inversion step

The first inversion step represents the core of the SBAS-InSAR inversion, and the second deconvolution is used to both optimize the input interferogram and estimate the deformation rate and residual topography for the first time prior to the next processing step^37^.

### Second inversion step

The second inversion step involves calculating the displacement of the time series based on the results of the first step. It is necessary to set the threshold for atmospheric filtering, the value of 1000 for spatial filtering, the value of 365 for temporal filtering, and the other program defaults in order to estimate and remove the atmospheric phase. In addition, the atmospheric influence is estimated so that it can be removed. During this step, there are four thresholds that are set after more than two experiments so as to determine the final threshold for the best processing results.

### Geocoding

Similar to the persistent scattering step, the geocoding step mainly involves geocoding the processing results obtained using the SBAS-InSAR technique. The results need to be displayed in color rendering by selecting the rainbow process in the change color table in order to display the different of surface settlement in different colors.

After this processing, it should be noted that the surface settlement area will be visualized and that the darker the color is, the greater the settlement amplitude.

## Comparison and analysis of the monitoring results

**Figure 9:**
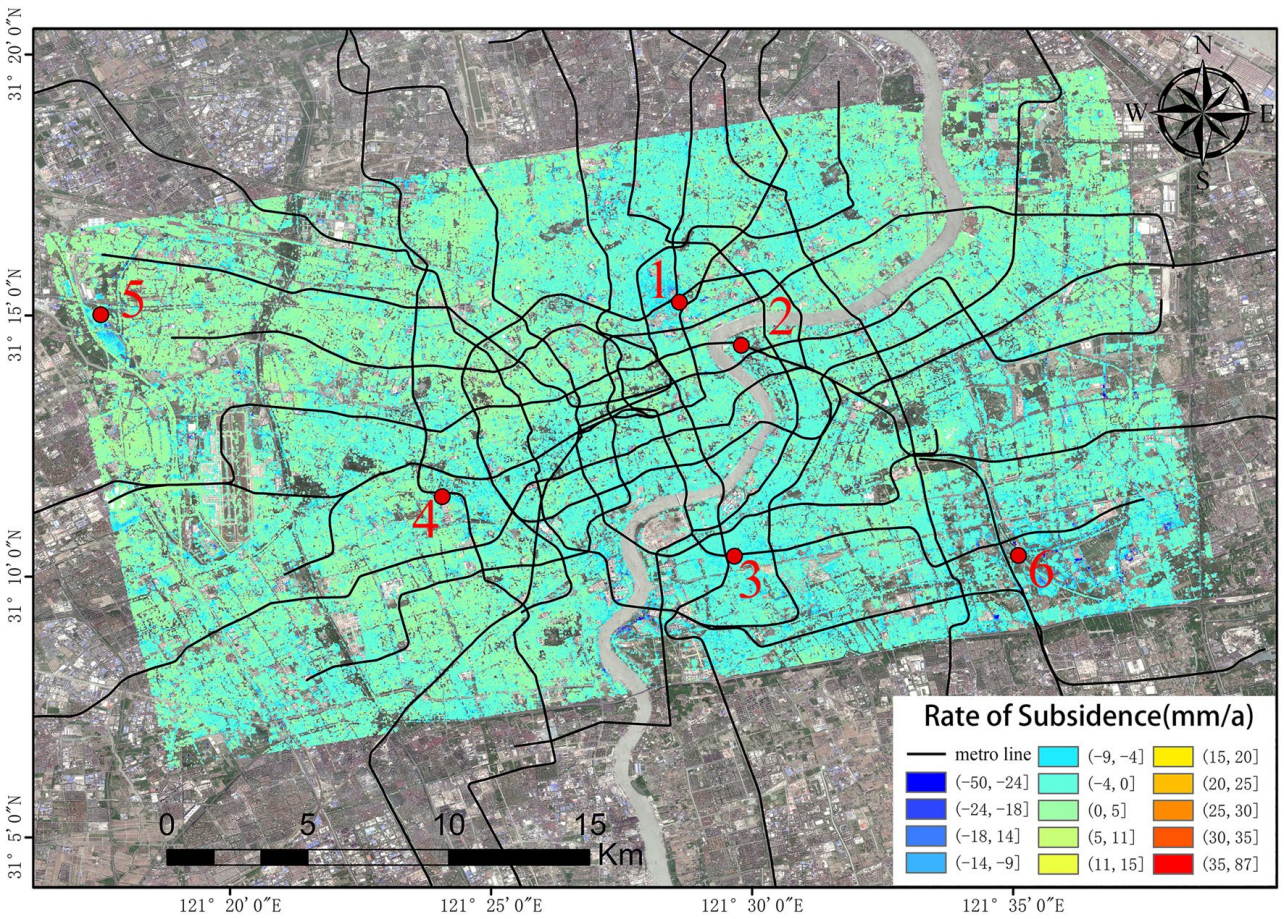
Ground subsidence rate map of Shanghai urban area (PS-InSAR). The Sentinel-1A and 2A have been acquired from the Alaska Satellite Center, https://vertex.daac.asf.alaska.edu/#. The data are visualized and integrated with SARScape 5.2.1 and ArcMap 10.5.0, http://www.esri.com.

**Figure 10:**
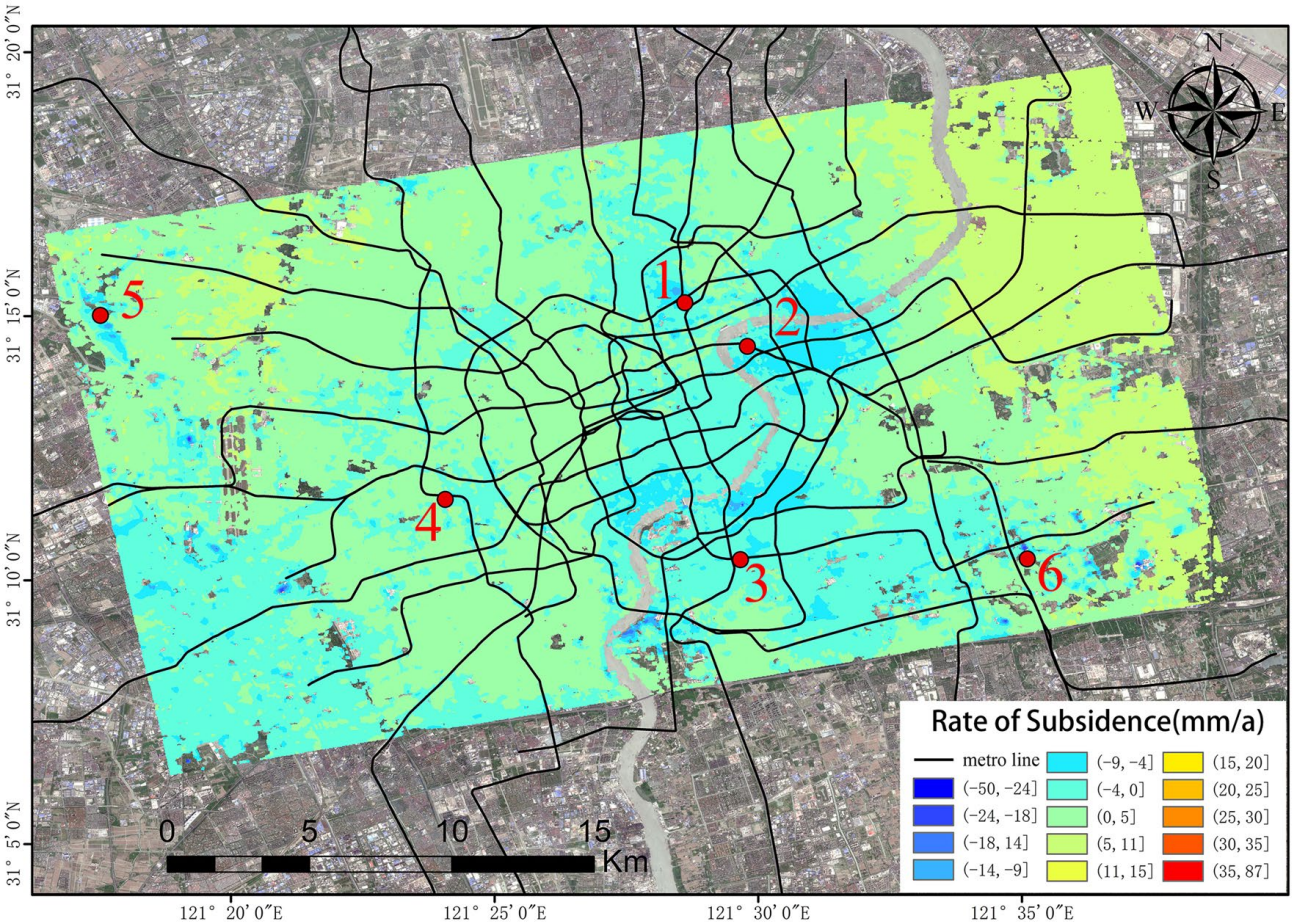
Ground subsidence rate map of Shanghai urban area (SBAS-InSAR). The Sentinel-1A and 2A has been acquired from the Alaska Satellite Center, https://vertex.daac.asf.alaska.edu/#. The data are visualized and integrated with SARScape 5.2.1 and ArcMap 10.5.0, http://www.esri.com.

The results concerning the settlement data from January 2019 to December 2020 in urban areas were obtained using two data processing procedures (PS-InSAR and SBAS-InSAR). The processing results obtained using the PS-InSAR technique are shown in Fig. 9, while the processing results obtained using the SBAS-InSAR technique are shown in Fig. 10.

The surface settlement rate maps processed on the basis of the SBAS-InSAR and PS-InSAR results indicate that the surface settlement in urban areas of Shanghai is not uniformly distributed, with several settlement funnels being apparent. A comparison of the monitoring results obtained using the two techniques reveals that the subsidence funnels shown by the SBAS-InSAR results are more obvious in Pingliang Road in Yangpu District, which makes up for the deficiency in the PS-InSAR monitoring results in an area with high vegetation coverage.

To determine the accuracy of the monitoring results, the results processed using the two technical methods need to be inter-comparison. Thus, six characteristic points within the settlement study area were selected for analysis, the specific locations of which are shown in Fig. 9. More specifically, feature site No. 1 is located near East Baoxing Road Station on Metro Line 3; No. 2 is located at Lujiazui Station on Metro Line 2 in Pudong New Area, which is in Shanghai’s central business district; No. 3 is located at Chengshan Road Station, which is at the intersection of Metro Line 8 and Metro Line 13; No. 4 is located on Metro Line 14 at Wuzhong Road Station; No. 5 is located at Hongqiao Railway Station; and No. 6 is located at the new campus of Shanghai University of Science and Technology.

New vector point layers were created using geographic information system (GIS) software to perform the vector annotation on the above-mentioned six feature points. The annotated vector point layers were then loaded into ENVI software, and the position information of each vector point used to extract the unit time information of the settlement result data processed using the PS-InSAR and SBAS-InSAR techniques.

**Figure 11:**
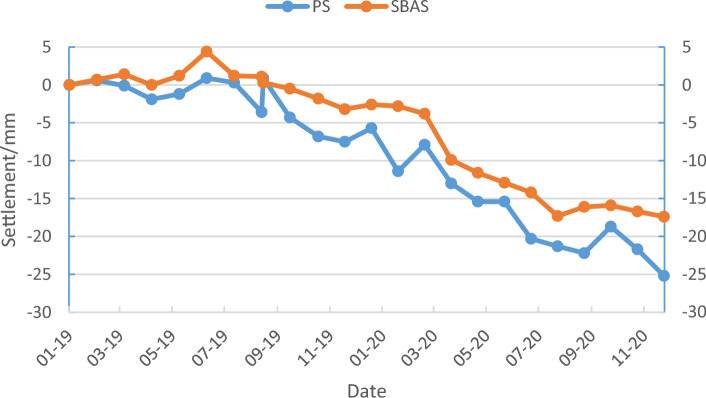
Settlement sequence of feature point No. 1 with PS and SBAS methods.

**Figure 12:**
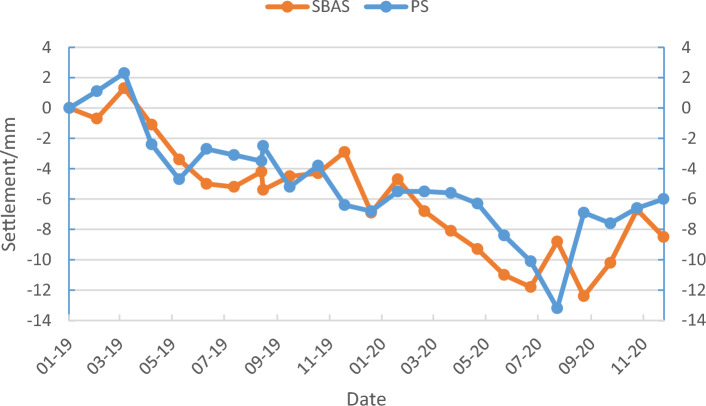
Settlement sequence of feature point 2 with PS and SBAS methods.

**Figure 13:**
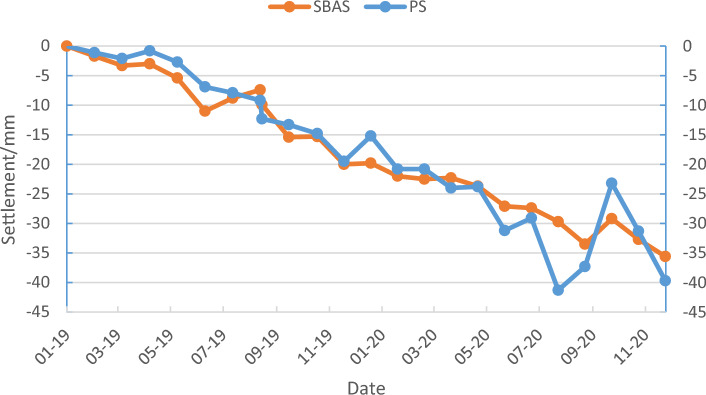
Settlement sequence of feature point 6 with PS and SBAS methods.

The monthly settlement data concerning the characteristic points within the urban surface settlement study area between January 2019 and December 2020 as obtained using the PS-InSAR and SBAS-InSAR techniques. To easily visualize the settlement of the feature points per unit time obtained using the two methods, three feature points were randomly selected to plot both the PS-InSAR and SBAS-InSAR results as a time-series line graph of the surface settlement (Figs. 11, 12, 13).

InSAR technology is used to measure ground deformation, including subsidence and uplift. In some cases, when the settlement reaches a certain level, the ground will rebound. From Figs. 11, 12 and 13, we can always see an occasional rebound in the ground elevation information at some monitoring sites, which may be caused by the following reasons:

1. *Human-caused* include filling and excavation, adding buildings, etc.
2. *Groundwater level change* If the groundwater level in a region decreases, settlement may occur. Once the groundwater level in the area rises again, the ground may rebound.
3. *Crustal adjustment* When an area experiences subsidence, the crust may adapt to the new gravity balance. This adjustment may cause the ground to rebound.
4. *Earthquake impact* If an earthquake occurs in an area, the surface deformation caused by the earthquake may lead to settlement. After an earthquake, the ground may also rebound.
5. *Rheological properties of rocks* Some rock flows have viscoelastic properties, and when they are compressed, they will settle. Once the pressure disappears, the rock flow quickly rebounds.
6. *Underground pumping* If large-scale underground pumping activities (such as oil or natural gas) are conducted in an area.

Through analysis, we believe that human activity and groundwater recharged may be the most dominant factor causing the ground rebound.

**Figure 14:**
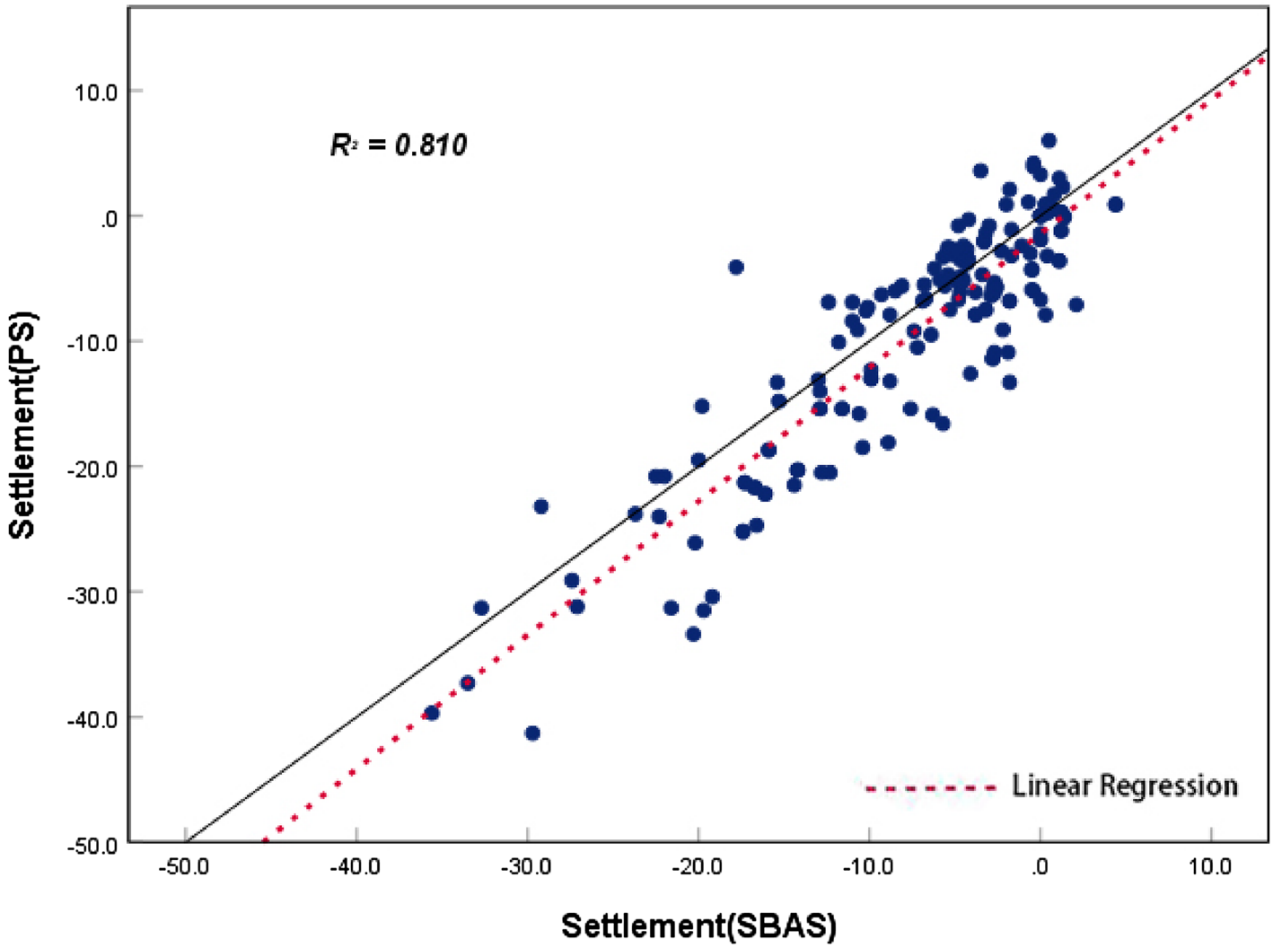
Regression analysis.

By observing the line graphs for the surface settlement per unit time at feature points No. 1, No. 2, and No. 6, it can be seen that the deformation of the surface settlement at all three feature points is basically the same at each time unit and that the change trends for all three exhibit a high level of consistency, which initially verifies the reliability of the PS-InSAR and SBAS-InSAR techniques. To further illustrate and verify the accuracy of the PS-InSAR method, a correlation analysis of the deformation around the characteristic points using two monitoring results per unit time was performed. The Pearson correlation analysis method was used to perform a correlation analysis of the six characteristic points, and the correlation analysis results are shown in Table 1, all the feature points from PS-InSAR and SBAS-InSAR were studied by using the regression analysis, the results of regression analysis for the sampling points show that there is a strong positive correlation between the monitoring results of SBAS-InSAR and PS-InSAR. It is evenly distributed near the fitting regression line (Fig. 14).

**Table 1:** Pearson correlation analysis results.

PS feature points	1	2	3	4	5	6
SBAS feature points	1	2	3	4	5	6
Pearson correlation coefficient	0.973	0.815	0.491	0.847	0.958	0.964

Based on the specification of the correlation analysis results, the values range from − 1 to 1. More specifically, between − 1 and 0, the two variables are negatively correlated; if the correlation coefficient is 0, the two variables are not correlated; between 0 and 1, the two variables are positively correlated; and the closer to 1, the higher the degree of correlation. According to the correlation analysis results presented, the correlation coefficients of feature points No. 1, No. 2, No. 4, No. 5, and No. 6 are all around 0.9, with the highest being 0.973 (indicating significant correlation at the 0.01 level), while feature point No. 3 has a lower correlation coefficient because the PS settlement point data are not covered and the adjacent data points are selected, although it does not affect the overall results of the correlation analysis. The correlation analysis revealed the results obtained using the two monitoring methods to be highly consistent, thereby fully demonstrating the feasibility and accuracy of the PS-InSAR technique.

## Analysis of the causes

**Figure 15:**
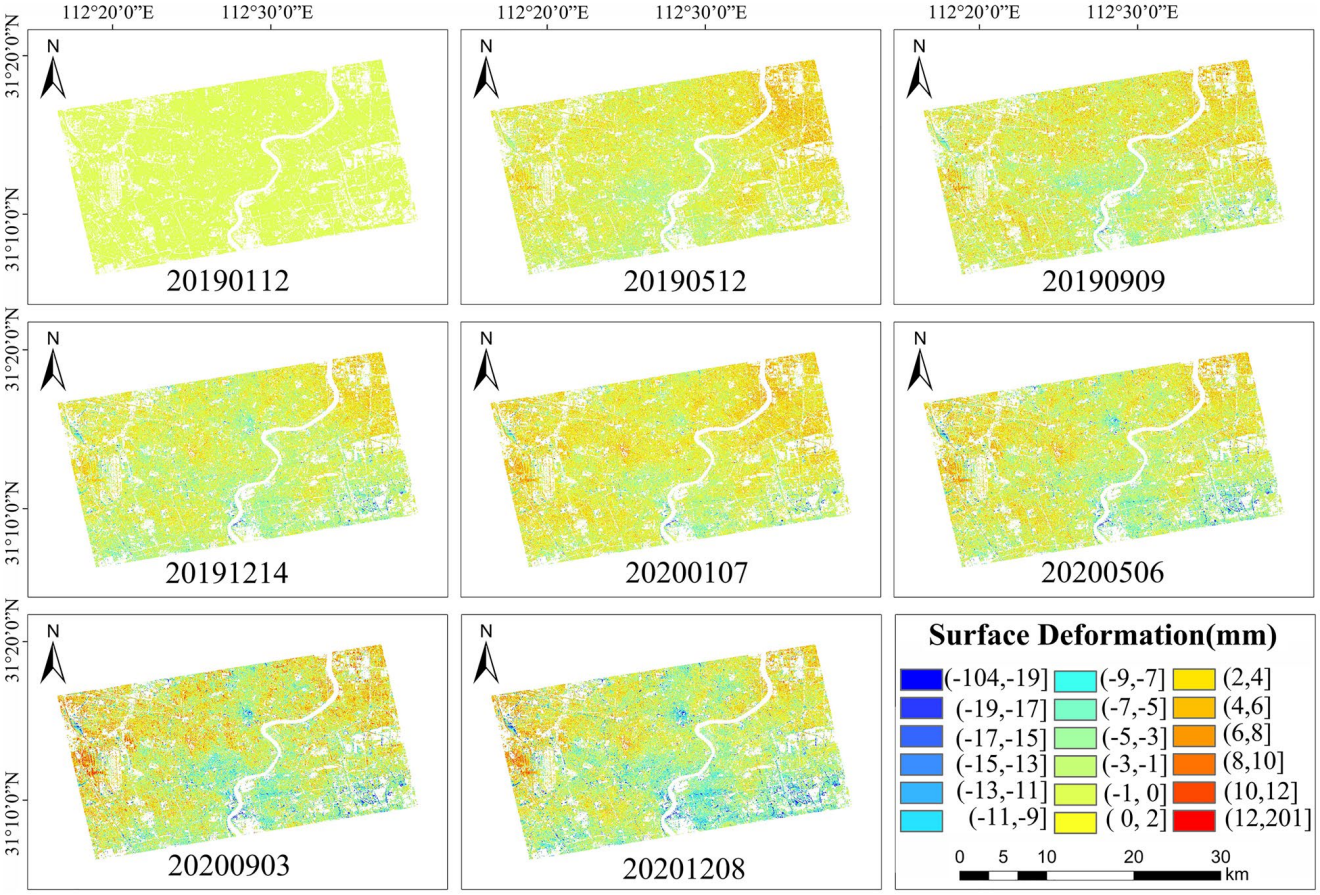
Time-series cumulative surface settlement. The Sentinel-1A has been acquired from the Alaska Satellite Center, https://vertex.daac.asf.alaska.edu/#. The data are visualized and integrated with SARScape 5.2.1 and ArcMap 10.5.0, http://www.esri.com.

The surface settlement data concerning urban areas of Shanghai cover 24 periods from January 2019 to December 2020. Based on the accumulated GS data, it is possible to obtain the change process in relation to the accumulated surface settlement within the study area, the magnitude of the change and the settlement trend, the distribution within the main settlement area, and the time-series map for the accumulated surface settlement within the study area, as shown in Fig. 15.

**Figure 16:**
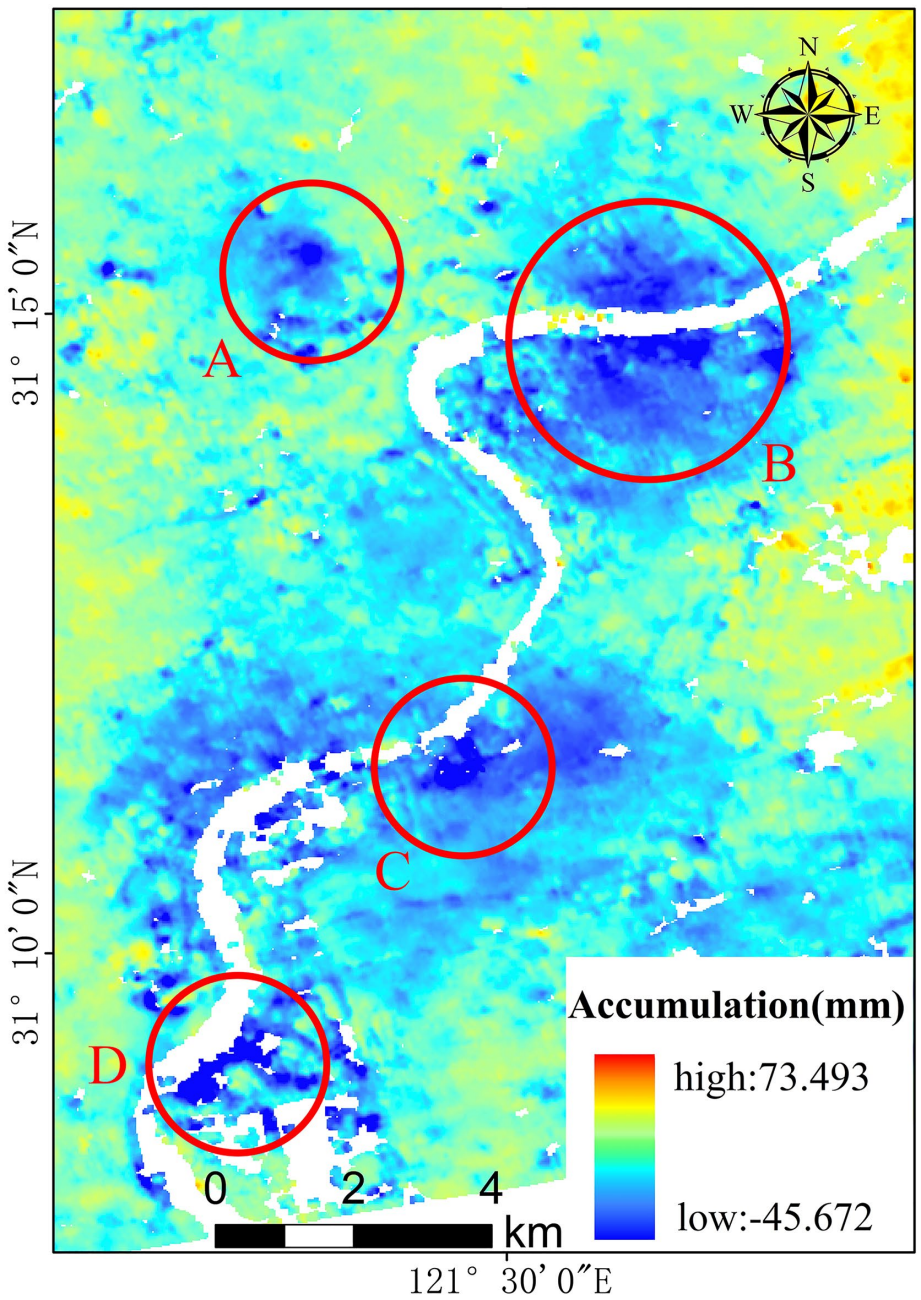
Settling funnel location. The Sentinel-1A has been acquired from the Alaska Satellite Center, https://vertex.daac.asf.alaska.edu/#. The data are visualized and integrated with SARScape 5.2.1 and ArcMap 10.5.0, http://www.esri.com.

According to the 24 maps of the time-series accumulated surface settlement, the surface is distributed unevenly. In light of the uneven surface settlement phenomenon, detailed maps of the settlement funnels were made, which revealed relatively obvious settlement funnels in Yangpu District, Hongkou District, Jing’an District, and part of Lujiazui in Pudong New Area (settlement funnels A and B). There were also obvious settlement funnels on both sides of the Huangpu River connected to Xuhui District and Pudong New Area (settlement funnels C and D), which settlement funnels A and B coincided with in terms of the historical settlement funnel areas within Shanghai. Based on historical data concerning surface settlement in Shanghai^6^, settlement funnel A in the Baoyuan Road area, which is located in Jing’an and Hongkou districts, is more consistent with the location of the settlement funnel identified in 1921 when surface settlement was found, while settlement funnel B in the Pingliang Road and Yangshupu Road area, which is located in Yangpu and Hongkou districts, has been recorded as surface settlement since the 1940s. The regional results regarding settlement funnels C and D coincide with the results concerning settlement funnels identified through ground subsidence monitoring from 2017 to 2019 using the SBAS-InSAR technique, with the time coinciding with this experiment conducted in 2019. Settlement funnels A, B, C, and D are shown in Fig. 16.

Feature point No. 1 is located within the area of settlement funnel A. The process and trend of the settlement change at point No. 1 can be obtained by performing a time-series analysis of the cumulative amount of settlement at feature point No. 1, which also represents the cumulative time-series change in settlement funnel A, as indicated in Fig. 16.

Based on data regarding the accumulated amount of GS at point No. 1, a time-series line graph is drawn showing the change in the accumulated amount at point No. 1, which reveals the magnitude and trend of the change in settlement at point No. 1.

According to above analysis, feature point No. 1 shows an overall trend of moderate and stable surface deformation from January 2019 to August 2019, while it shows an overall trend of continuous subsidence from September 2019 to December 2020. In fact, the annual settlement rate reaches 20.00 mm/year, which indicates a high rate of surface settlement. The accumulated surface settlement at point No. 1 reaches 25.20 mm in December 2020. The maximum surface settlement in the study area reaches approximately 90 mm according to the settlement monitoring data.

Such a high rate of GS is closely related to the geological conditions of Shanghai, which is located in a coastal area of the Yangtze River Delta, a late land-forming area. Overall, the terrain within the urban area is flat. With an average elevation of 3–4.5 m, it is a relatively typical soft soil type area. According to Table 2^14^, in terms of the overall geological condition of Shanghai, the soft soil geological type is influenced by the city’s municipal and other engineering^53^. Indeed, the rapid urbanization of Shanghai, the construction of urban buildings, both aboveground and underground transportation pipelines and other facilities have increased the surface load, which has led to the deformation of the soft soil layer and resulted in disaster phenomena such as surface subsidence. Shanghai is very rich in groundwater resources. Due to the rapid increase in the city’s population, the excessive extraction can damage the water content of the underground soil layer, which would lead to the destruction of the soil layer, resulting in surface deformation following the weakening of the surface-bearing capacity. As early as the 1990s, Shanghai suffered severe land subsidence due to this reason. Now, this type of ground subsidence has been prevented or mitigated by recharging groundwater.

**Table 2:** Summary of geological information of Shanghai area.

Geological formations	Soil type	Thickness (m)	Top burial depth (m)	Hydrogeology
Topsoil	Clay	1.5–4.0	0.5–2.0	
First sandy soil layer	Silty chalk	3.0–20.0	2.0–3.0	Submerged layer
First soft soil layer	Silty, powdery clay Soft clay	5.0–20.0	3.0–15.0	
Second soft soil layer	Soft clay, clay Chalky sand	10.0–25.0	15.0–20.0	Micropressurized aquifers
First hard soil layer	Hard clay	1.5–6.0	20.0–30.0	
Second sandy soil layer	Silty chalky sand Silty fine sand	10.0–22.0	28.0–35.0	First aquifer
Third soft soil layer	Clay with chalk	20.0–40.0	40.0–50.0	
Third sandy soil layer	Clay-bearing chalky sand Fine sand, gravelly fine sand	20.0–60.0	61.0–77.0	Second aquifer

## Conclusion

In this paper, based on the PS-InSAR and SBAS-InSAR techniques, 24 images of Sentinel-1A data concerning areas of Shanghai were used as the experimental area to experimentally investigate the surface settlement, obtain a surface settlement map, and then verify and analyze the causes of the settlement. Given the amount per unit time of surface settlement at the selected characteristic points, the results show that the morphological variables relevant to surface settlement remain basically consistent at all time points and that their change trends display a high level of agreement with the monitoring results, which verifies the reliability of the PS-InSAR monitoring method. Moreover, the PS-InSAR and SBAS-InSAR results are inter-compared and the monitoring results found to be highly consistent, which indicates the reliability of PS-InSAR monitoring.

By analyzing the subsidence rate and accumulated amount of subsidence based on the monitoring results, the urban area of Shanghai is mainly characterized by an uneven surface, with the settlement and multiple subsidence funnels being distributed across the main urban area. Through a comparison with historical settlement data, geological data, and urban construction distribution data, the individual settlement funnels can be seen to correspond with the historical surface settlement funnel data, while the settlement funnels are mainly distributed in areas such as the intersection sites of subway lines. Therefore, the surface settlement in urban areas in Shanghai exhibits some degree of correlation with the city’s geological environment, human construction activities, and ground load.

Moverover, DEM is a crucial input parameter in the processing of InSAR data. This is because InSAR data is acquired by reflecting radar signals off ground targets and then computing ground elevation changes from the phase information of multiple radar data. To achieve this, DEM is used to convert the interference phase into ground elevation changes. If the DEM’s accuracy is not high enough, the calculated ground elevation changes will be inaccurate, which may mask the true surface change signal or generate misinterpreted surface change signals. Therefore, high-quality DEM must be used to obtain accurate results in InSAR data processing. Furthermore, the accuracy of DEM also affects the accuracy and reliability of surface deformation fields. The higher the accuracy of the DEM, the more accurately it can calculate the surface deformation field, thereby improving the reliability and accuracy of the results. In future studies, high-precision DEM will be combined with InSAR technology to improve the accuracy and efficiency of ground subsidence monitoring, meanwhile, we can measure some points in the study area to verify the InSAR results on the spot.

## Data Availability

All raw image data are derived from https://vertex.daac.asf.alaska.edu/#. The datasets generated during and/or analysed during the current study are available from the corresponding author on reasonable request.

## References

[1] Huang S, Li X, Wang Y (2012). A new model of geo-environmental impact assessment of mining: A multiple-criteria assessment method integrating Fuzzy-AHP with fuzzy synthetic ranking. Environ. Earth Sci..

[2] Gerardo HG, Pablo E, Tomas R (2021). Mapping the global threat of land subsidence. Science.

[3] Fentahun TM, Bagyaraj M, Melesse MA (2021). Seismic hazard sensitivity assessment in the Ethiopian Rift, using an integrated approach of AHP and DInSAR methods. Egypt. J. Remote Sens. Space Sci..

[4] Greif V, Vlcko J (2012). Monitoring of post-failure landslide deformation by the PS-InSAR technique at Lubietova in Central Slovakia. Environ. Earth Sci..

[5] Zhang J, Feng DX, Qi W (2018). Monitoring land subsidence in Panjin region with SBAS-InSAR method. J. Eng. Geol..

[6] Shi, Y. J. Recent characteristics of land subsidence in Shanghai and its effect on performance of key municipal facilities. Shanghai Jiao Tong University (2018).

[7] Zhang ZH, Hu CT, Zhang Z (2022). PS-InSAR based monitoring and analysis of surface subsidence in Shanghai. Remote Sens. Nat. Resour..

[8] Zhu L, Gong HL, Li XJ, Wang R, Chen BB, Dai ZX, Teatini P (2015). Land subsidence due to groundwater withdrawal in the northern Beijing plain, China. Eng. Geol..

[9] Zhou C, Gong B, Chen W, Li M, Gao F, Zhu W, Liang Y (2017). InSAR time-series analysis of land subsidence under different land use types in the eastern Beijing Plain, China. Remote Sens..

[10] Lucia S, Federico A, Gabriele N, Marco V, Beniamino M (2020). Early estimation of ground displacements and building damage after seismic events using SAR and LiDAR data: The case of the Amatrice earthquake in central Italy, on 24th August 2016. Int. J. Disaster Risk Reduct..

[11] Thomas AV, Saha S, Danumah JH (2021). Landslide susceptibility zonation of Idukki district using GIS in the Aftermath of 2018 Kerala floods and landslides: A comparison of AHP and frequency ratio methods. J. Geovisualization Spatial Anal..

[12] Azarakhsh Z, Azadbakht M, Matkan A (2022). Estimation, modeling, and prediction of land subsidence using Sentinel-1 time series in Tehran-Shahriar plain: A machine learning-based investigation. Remote Sens. Appl. Soc. Environ..

[13] Ghasemloo N, Matkan AA, Alimohammadi A (2022). Estimating the agricultural farm soil moisture using spectral indices of Landsat 8, and Sentinel-1, and artificial neural networks. J. Geovisualization Spatial Anal..

[14] Shanghai Municipal Bureau of Statistics. Shanghai Statistical Yearbook 2020 (China Statistics Press, 2020).

[15] Yang Q, Ke YH, Zhang DY, Chen BB, Gong HL, Lv MY, Zhu L, Li XJ (2018). Multi-scale analysis of the relationship between land subsidence and buildings: A case study in an eastern Beijing urban area using the PS-InSAR technique. Remote Sens..

[16] Lyu MY, Ke YH, Guo L (2020). Change in regional land subsidence in Beijing after south-to-north water diversion project observed using satellite radar interferometry. GI Sci. Remote Sens..

[17] Yan Y (2020). Ground Subsidence Monitoring and Mechanism Analysis in Haikou Area Based on SBAS-InSAR Technology.

[18] Nie YJ, Liu GX, Jin SF (2013). Ground subsidence of shanghai from 2009 to 2010 monitored by PS-InSAR technique. Remote Sens. Inf..

[19] Xiong ST, Wang CS, Qin XQ (2021). Time-series analysis on persistent scatter-interferometric synthetic aperture radar (PS-InSAR) derived displacements of the Hong Kong–Zhuhai–Macao Bridge (HZMB) from Sentinel-1A observations. Remote Sens..

[20] Gao ET, Fan DL, Fu BL (2019). Land subsidence monitoring of Nanjing area based on PS-InSAR and SBAS technology. Geod. Geodyn..

[21] Xong JC, Nie YJ, Luo Y (2019). Monitoring urban land subsidence by dual-polarization Sentinel-1data: A case study of Shanghai. Bull. Surv. Mapp..

[22] Calais E, Dong L, Wang M, Shen Z, Vergnolle M (2006). Continental deformation in Asia from a combined GPS solution. Geophys. Res. Lett..

[23] Kumar K, Pant MC, Satyal GS, Dumka RK (2008). Comparison of digital surface modeling techniques for sloping hill terrain using GPS data. Int. J. Model. Simul..

[24] Ahmed R, Mahmud KH, Tuya JH (2021). A GIS-based mathematical approach for generating 3d terrain model from high-resolution UAV imageries. J. Geovisualization Spatial Anal..

[25] Pu CH, Xu Q, Zhao KY (2021). Land uplift monitoring and analysis in Yan’an new district based on SBAS⁃InSAR technology. Geomat. Inf. Sci. Wuhan Univ..

[26] Chen M, Tomás R, Li ZH, Motagh M, Li T, Hu LY, Gong HL, Li XJ, Yu J, Gong XL (2016). Imaging land subsidence induced by groundwater extraction in Beijing using satellite radar interferometry. Remote Sens..

[27] Fuhrmann T, Garthwaite MC (2019). Resolving three-dimensional surface motion with InSAR: Constraints from multi-geometry data fusion. Remote Sens..

[28] Xu Q, Pu CH, Zhao KY, He P, Zhang HY, Liu JL (2020). Time series InSAR monitoring and analysis of spatiotemporal evolution characteristics of land subsidence in Yan'an New District. Geomat. Inf. Sci. Wuhan Univ..

[29] Dumka RK, SuriBabu D, Malik K, Prajapati S (2020). PS-InSAR derived deformation study in the Kachchh, Western India. Appl. Comput. Geosci..

[30] Ferretti A, Prati C, Rocca F (2001). Permanent scatterers in SAR interferometry. IEEE Trans. Geosci. Remote Sens..

[31] Zhang ZJ (2016). Research on Settlement Monitoring of High Speed Railway Based on PS-InSAR Technology.

[32] Berardino P, Fornaro G, Lanari R, Sansosti E (2002). A new algorithm for surface deformation monitoring based on small baseline differential SAR interferograms. IEEE Trans. Geosci. Remote Sens..

[33] Mohammadimanesh F, Salehi B, Mahdianpari M, English J, Chamberland J, Alasset P (2019). Monitoring surface changes in discontinuous permafrost terrain using small baseline SAR interferometry, object-based classification, and geological features: A case study from Mayo, Yukon Territory, Canada. GI Sci. Remote Sens..

[34] Liu X, Wang P, Lu Z, Gao K, Wang H, Jiao C, Zhang X (2019). Damage detection and analysis of urban bridges using terrestrial laser scanning (TLS), ground-based microwave interferometry, and permanent scatterer interferometry synthetic aperture radar (PS-InSAR). Remote Sens..

[35] Riccardo L, Francesco C, Mariarosaria M (2007). An overview of the small baseline subset algorithm: A DInSAR technique for surface deformation analysis. Pure Appl. Geophys..

[36] Federico R, Francesco C, Matteo DS (2022). Review of satellite radar interferometry for subsidence analysis. Earth-Sci. Rev..

[37] Zhou CF, Gong HL, Chen BB, Zhu F, Duan GY, Gao ML, Lu W (2016). Land subsidence under different land use in the eastern Beijing plain, China 2005–2013 revealed by InSAR timeseries analysis. GI Sci. Remote Sens..

[38] Ma YY, Zou XQ, Ma WF (2019). Settlement monitoring and analysis of Tianjin area based on PS-InSAR. Remote Sens. Technol. Appl..

[39] Liu X, Shang AR (2016). Application contrast of PS-InSAR and SBAS-InSAR in urban surface subsidence monitoring. GNSS World China.

[40] Cigna F, Tapete D (2021). Present-day land subsidence rates, surface faulting hazard and risk in Mexico City with 2014–2020 Sentinel-1 IW InSAR. Remote Sens. Environ..

[41] Francesca C, Deodato T (2021). Satellite InSAR survey of structurally-controlled land subsidence due to groundwater exploitation in the Aguascalientes Valley, Mexico. Remote Sens. Environ..

[42] Khorrami M, Abrishami S, Maghsoudi Y (2020). Extreme subsidence in a populated city (Mashhad) detected by PSInSAR considering groundwater withdrawal and geotechnical properties. Sci. Rep..

[43] Maghsoudi Y, Amani R, Ahmadi H (2021). A study of land subsidence in west of Tehran using Sentinel-1 data and permanent scatterer interferometric technique. Arab. J. Geosci..

[44] Mahmoodinasab F, Mohseni N (2021). A spatiotemporal analysis of the relationship between groundwater level and ground surface displacement using Sentinel-1 SAR data. Arab. J. Geosci..

[45] Espiritu KW, Reyes CJ, Benitez TM (2022). Sentinel-1 Interferometric Synthetic Aperture Radar (InSAR) reveals continued ground deformation in and around Metro Manila, Philippines, associated with groundwater exploitation. Nat. Hazards.

[46] Yang CS, Lu Z, Zhang Q, Liu RC, Ji LY, Zhao CY (2019). Ground deformation and fissure activity in Datong basin, China 2007–2010 revealed by multi-track InSAR. Geomat. Nat. Hazards Risk.

[47] Yao S, He Y, Zhang L, Yang W, Chen Y, Sun Q, Zhao Z, Cao S (2023). A convLSTM neural network model for spatiotemporal prediction of mining area surface deformation based on SBAS-InSAR monitoring data. IEEE Trans. Geosci. Remote Sens..

[48] He Y, Yan H, Yang W, Yao S, Zhang L, Chen Y, Liu T (2022). Time-series analysis and prediction of surface deformation in the Jinchuan mining area, Gansu Province, by using InSAR and CNN–PhLSTM network. IEEE J. Sel. Top. Appl. Earth Obs. Remote Sens..

[49] Zhang ZH, Zhang SB, Hu CT (2023). Hazard assessment model of ground subsidence coupling AHP, RS and GIS—A case study of Shanghai. Gondwana Res..

[50] Sousa JJ, Ruiz AM, Hanssen RF (2010). PS-InSAR processing methodologies in the detection of field surface deformation-Study of the Granada basin (Central Betic Cordilleras, southern Spain). J. Geodyn..

[51] Luo SM, Du KF, Chang L (2014). Ground subsidence rates of beling area inversed by PS-InSAR analysis. J. Geod. Geodyn..

[52] Zhang ZH, Wu XW, Li YK (2022). Monitoring environment transformation along the BTIC railway based on remote sensing by utilizing the R_RSEI. Photogramm. Eng. Remote Sens..

[53] Wu SB, Yang ZF, Ding XL, Zhang BC, Zhang L, Lu Z (2020). Two decades of settlement of Hong Kong international airport measured with multi-temporal InSAR. Remote Sens. Environ. Interdiscip. J..

